# Role of Hippo pathway dysregulation from gastrointestinal premalignant lesions to cancer

**DOI:** 10.1186/s12967-024-05027-8

**Published:** 2024-02-29

**Authors:** Giulia Schiavoni, Beatrice Messina, Stefano Scalera, Lorenzo Memeo, Cristina Colarossi, Marzia Mare, Giovanni Blandino, Gennaro Ciliberto, Giulia Bon, Marcello Maugeri-Saccà

**Affiliations:** 1 Clinical Trial Center, Biostatistics and Bioinformatics Unit, Department of Research, Diagnosis and Innovative Technologies, IRCCS Regina Elena National Cancer Institute, Rome, Italy; 2 SAFU Laboratory, Department of Research, Advanced Diagnostic, and Technological Innovation, IRCCS Regina Elena National Cancer Institute, Rome, Italy; 3 Pathology Unit, Mediterranean Institute of Oncology, Viagrande, Italy; 4 Medical Oncology Unit, Mediterranean Institute of Oncology, Viagrande, Italy; 5 Department of Biomedical, Dental, Morphological and Functional Imaging Sciences, University of Messina, Messina, Italy; 6 Translational Oncology Research Unit, Department of Research, Diagnosis and Innovative Technologies, IRCCS Regina Elena National Cancer Institute, Rome, Italy; 7 Scientific Directorate, IRCCS Regina Elena National Cancer Institute, Rome, Italy; 8 Cellular Network and Molecular Therapeutic Target Unit, Department of Research, Diagnosis and Innovative Technologies, IRCCS Regina Elena National Cancer Institute, Via Elio Chianesi 53, 00144 Rome, Italy; 9 Division of Medical Oncology 2, IRCCS Regina Elena National Cancer Institute, Rome, Italy

**Keywords:** Hippo pathway, YAP/TAZ, Gastrointestinal tumors, Precancerous lesions, Chronic inflammation, Fibrosis

## Abstract

**Background:**

First identified in *Drosophila melanogaster*, the Hippo pathway is considered a major regulatory cascade controlling tissue homeostasis and organ development. Hippo signaling components include kinases whose activity regulates YAP and TAZ final effectors. In response to upstream stimuli, YAP and TAZ control transcriptional programs involved in cell proliferation, cytoskeletal reorganization and stemness.

**Main text:**

While fine tuning of Hippo cascade components is essential for maintaining the balance between proliferative and non-proliferative signals, pathway signaling is frequently dysregulated in gastrointestinal cancers. Also, YAP/TAZ aberrant activation has been described in conditions characterized by chronic inflammation that precede cancer development, suggesting a role of Hippo effectors in triggering carcinogenesis. In this review, we summarize the architecture of the Hippo pathway and discuss the involvement of signaling cascade unbalances in premalignant lesions of the gastrointestinal tract, providing a focus on the underlying molecular mechanisms.

**Conclusions:**

The biology of premalignant Hippo signaling dysregulation needs further investigation in order to elucidate the evolutionary trajectories triggering cancer inititation and develop effective early therapeutic strategies targeting the Hippo/YAP pathway.

## Introduction

Nearly three decades of intense research have established the involvement of the Hippo pathway and its effectors Yes-associated protein (YAP) and the paralog transcriptional co-activator with PDZ-binding domain (TAZ) in shaping organ size control, tissue homeostasis, stem cell fate and cancer [1]. Early studies conducted in fly models [2, 3] together with evidence from mice have been instrumental in delineating the structural and functional organization of the pathway and revealing its evolutionarily conserved nature. Overall, these studies helped to clarify the sequence according to which the various components interact along the pathway and the associated gene expression [4]. The Hippo pathway is organized in a signaling cascade of serine-threonine kinases and adaptors that ultimately inhibit the nuclear translocation of YAP/TAZ transcriptional co-factors. Conversely, when the signaling cascade is off, YAP/TAZ translocate to the nucleus and interact with TEA domain-containing sequence-specific transcription factors (TEAD1 to TEAD4), thus modulating the transcription of specific target genes [5].

The Hippo pathway is considered a tumor-suppressor cascade, as signaling dysregulation resulting in YAP/TAZ aberrant activation fuels tumor onset and progression [1, 6, 7]. High-throughput approaches have helped to outline the specific transcriptional programs dictated by YAP/TAZ activity, revealing the regulation of a variety of cancer-related cellular processes, such as invasion/metastatic dissemination [5], stemness [7, 8] and chemoresistance [9].

Tumors of the gastrointestinal (GI) tract are a group of malignancies including colorectal cancer (CRC), hepatocellular carcinoma (HCC), pancreatic cancer (PDAC), gastric cancer (GC) and esophageal cancer (EC), with an overall estimated global incidence of 26% [10]. The first evidence tying Hippo to oncogenesis described the onset of liver tumors following transgenic YAP overexpression in mouse hepatocytes [11]. Since then, many efforts have been made to elucidate the mechanisms through which Hippo pathway dysregulations lead to the onset of GI cancers and contribute to disease progression. Evidence collected from these studies suggest an early involvement in tumor initiation and even before, in the pathogenesis of premalignant conditions. Firstly, YAP/TAZ high expression have been observed early during carcinogenesis and in cancer-predisposing diseases [12–16]. Then, studies in mouse models outlined the oncogenic potential of tissue regeneration programs controlled by YAP/TAZ. Prolonged stimuli might unbalance Hippo signaling towards YAP-pro-proliferative outputs linking YAP to carcinogenesis [17, 18]. Finally, the Hippo pathways is a crucial regulator of the immune system and an early dysregulation holds the potential to fuel carcinogenesis through multi-faceted mechanisms including impairment of immune responses [19].

In this review, we address the involvement of Hippo pathway dysregulation in premalignant diseases of CRC, HCC, PDAC and EC and in their eventual progression to malignancy. Hippo dysregulation in GC development has been thoroughly addressed elsewhere [12]. We focus on the mechanisms promoting early Hippo pathway dysregulation in inflammation, fibrosis and chronic conditions induced by prolonged damage in the GI tract and discuss the related clinical implications.

## The network of Hippo pathway

**Fig. 1:**
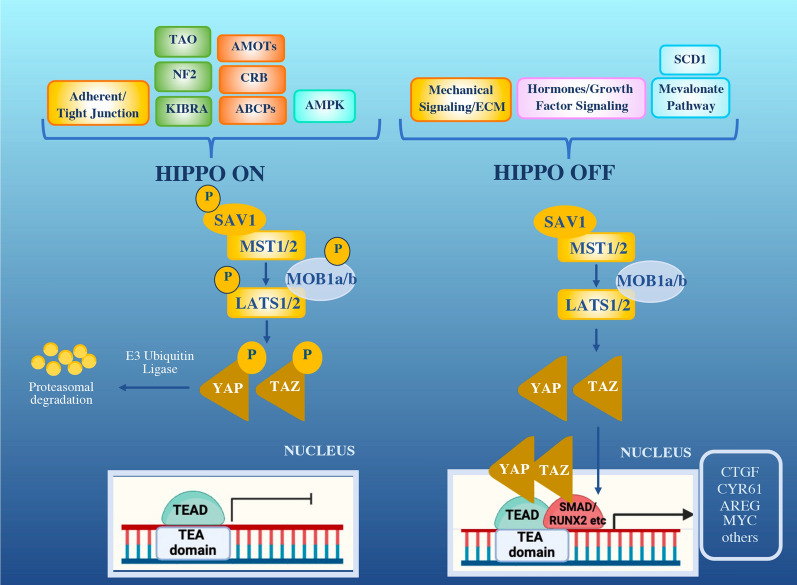
Hippo signaling pathway and its network. When upstream signals activate the Hippo pathway (Hippo ON), the phosphorylation cascade is enabled that ultimately induces YAP/TAZ proteasomal degradation (leftside). In Hippo OFF conformation, promoted by mechanical signaling and hormones, YAP/TAZ can translocate to the nucleus. Here, by interacting with TEAD transcription factors and cofactors, YAP/TAZ activate the transcription of target genes. ABCP: apico-basal cell polarity protein; AMOT: angiomotin; AMPK: AMP-activated protein kinase; AREG: amphiregulin; CTGF: connective tissue growth factor; CRB: crumbs; CYR61: Cysteine-rich angiogenic inducer 61; KIBRA: kidney and brain expressed protein; NF2: neurofibromin 2; RUNX2: Runt-related transcription factor 2; SCD1: Stearoyl-CoA-desaturase-1; SMAD: small mother against decapentaplegic; TAO: thousand-and-one amino acid

The Hippo regulatory module contains the serine/threonine kinases sterile 20-like kinase 1 and 2 (MST1/2) and large tumor suppressor 1 and 2 (LATS1/2), along with the scaffold proteins Salvador homolog 1 (SAV1) and MOB kinase activator 1A and 1B (MOB1A/B) [1]. The signaling cascade modulated by this set of kinases and adaptors induce the phosphorylation of the Hippo transducers YAP and TAZ, promoting their cytoplasmic retention and proteasomal degradation (Fig. 1) [5].

Several stimuli regulate Hippo signaling cascade and operate through different mechanisms, in a context-specific manner. Schematically, these processes can be grouped in: (i) upstream mechanical cues [20]; (ii) hormones and growth factors activating G-protein-coupled receptors (GPCRs) and Rho GTPases [21]; and (iii) metabolic pathways [22, 23], cellular energy sensor AMP-activated protein kinase (AMPK) [24], and Stearoyl-CoA desaturase-1 (SCD1) fatty acid pathways [25].

In tumors, YAP/TAZ hyperactivation is frequent and may result from dysfunctional Hippo signaling and/or upstream stimuli that bypass Hippo kinases [26]. In this context, YAP/TAZ are accumulated into the nucleus, interact with TEADs or other transcriptional partners (SMADs, TBX5, RUNX1/2) and induce the transcription of target genes [7, 27]. Besides cancer-related proteins, YAP/TAZ-TEAD targets also include negative pathway regulators and ligands mediating the activity of other pathways [Sonic Hedgehog (SHH), Wnt/β-catenin, transforming-growth factor β (TGF-β) and NOTCH], thus indicating the existence of both autoregulatory feedback loops and crosstalk with stem-cell pathways [28–31].

## Hippo pathway dysregulation on the way to liver carcinogenesis

In normal liver, the Hippo pathway plays a pivotal role in shaping morphology and controlling hepatocytes proliferation. Pioneering studies conducted in mice elucidated how the pathway and its effectors coordinately act to ensure liver homeostasis. While under physiological conditions the differentiation status of mouse hepatocytes is finely controlled by the Hippo pathway, YAP overexpression promoted dedifferentiation of liver progenitor cells [32]. The resulting increase in liver mass could be reverted upon YAP reduction [33]. Although reversible, hepatomegaly caused by continuous YAP overexpression finally led to the formation of nodules harboring the features of HCC, in a process that required YAP-TEAD interaction [13]. Hepatomegaly and eventually HCC have also been observed in WW45 (the mouse homolog of SAV1) knock-out (KO) mice [34]. Moreover, liver-specific KO of Hippo regulatory genes yielded similar outcomes [34–38].

YAP/TAZ overexpression and/or signatures denoting their activity have been associated with aggressive molecular features and poor survival outcomes in HCC patients [39, 40]. YAP/TAZ control HCC progression through multiple mechanisms including crosstalk with protumorigenic pathways [41–45] and interaction with metabolic processes [46, 47] and stemness factors [48, 49]. In addition, Hippo pathway dysregulation is involved in resistance to a variety of treatments directed to liver cancer [50–52].

**Fig. 2:**
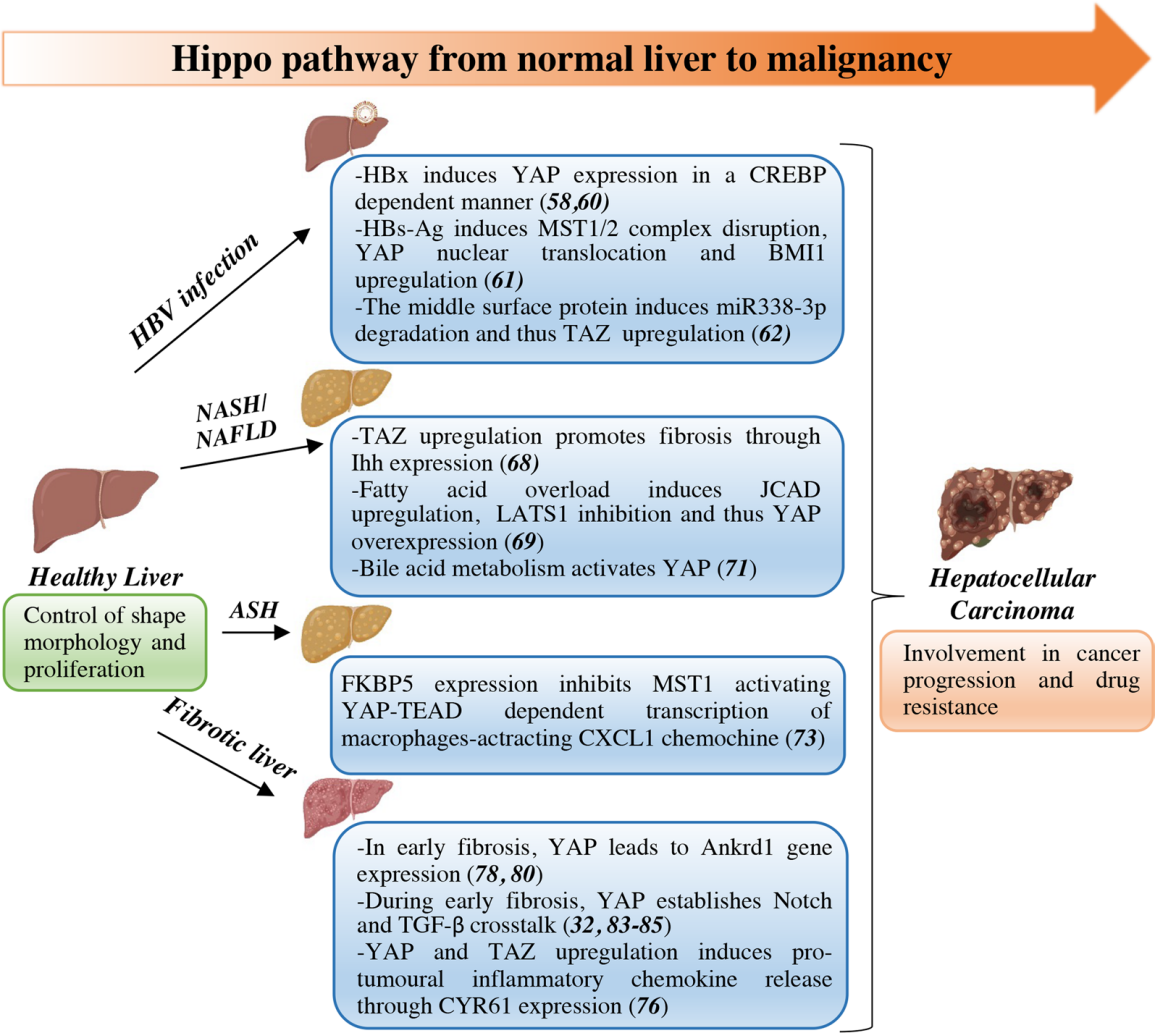
Hippo pathway dysregulation from liver precursor lesions to malignancy*.* Mechanisms contributing to YAP/TAZ aberrant activation in hepatic precancerous lesions (HBV infection, NAFLD/NASH/ASH and liver fibrosis). ANKRD11: cardiac ankyrin repeat protein; ASH: alcoholic steatohepatitis; BMI1: B lymphoma Mo-MLV insertion region 1 homolog; CREBP: cAMP response element-binding protein; CXCL1: CXC motif chemokine ligand 1; CYR61: cysteine-rich angiogenic inducer 61; FKBP5: FK506-binding protein 51;HBsAg: HBV surface antigen; HBV: hepatitis B virus; HBx: hepatitis B virus X protein; HCC: hepatocellular carcinoma; IHH: Indian hedgehog; JCAD: junctional cadherin 5 associated; LATS1: large tumor suppressor 1; MST1/2: serine/threonine kinases sterile 20-like kinase 1 and 2; NAFLD: nonalcoholic fatty liver disease; NASH: nonalcoholic steatohepatitis; NOTCH: neurogenic locus notch homolog protein; TAZ: Transcriptional co-activator with PDZ-binding domainTEAD: TEA domain-containing sequence-specific transcription factors; TGF-β**:** transforming growth factor beta; YAP: Yes-associated protein

Concordant with early YAP activation in liver cancer development, the etiology of liver tumorigenesis is connected with a multitude of Hippo-affecting cues. Established risk factors are hepatitis B virus (HBV)/hepatitis C virus (HCV) chronic infection, alcoholic/nonalcoholic steatohepatitis (ASH/NASH) and nonalcoholic fatty liver disease (NAFLD), metabolic (diabetes and obesity) and lifestyle (smoking) factors [53]. These conditions all result in chronic liver disease, usually accompanied by liver fibrosis and cirrhosis, which has been estimated to contribute to approximately 90% of all HCC cases [54]. Evidence of dysfunctional Hippo signaling linking precancerous chronic disease to cancer is discussed below and summarized in Fig. 2.

### Hippo pathway and HCV/HBV infection

Among risk factors associated with HCC development, hepatitis virus infections play a major role, with HBV and HCV detected in 80% of patients [55]. However, the risk linked to HBV/HCV infection has decreased over the last two decades due to neonatal HBV vaccination and availability of effective antiviral drugs [56, 57].

Various mechanisms contribute to YAP activation in HBV-infected liver. HBV X protein (HBx), an established driver of HBV-mediated chronic disease and liver cancer, transcriptionally induces YAP in a cyclic adenosine monophosphate (cAMP) response element-binding protein (CREB)-dependent manner. Accordingly, YAP expression levels were dramatically increased in HBV-infected hepatoma cells and in the liver of HBx transgenic mice [58], while immunohistochemical YAP expression/nuclear accumulation and HBx expression were correlated in HBV-positive HCC samples [59]. High YAP levels are further ensured by E3 ligase HDM2-dependent NEDDylation of HBx promoting its stability [60]. In HBV surface antigen (HBsAg)-transgenic mice, Hippo signaling disruption by MST1/2 inactivation caused the nuclear translocation of YAP and upregulation of BMI1 proto-oncogene resulting in sustained hepatocarcinogenesis. Treatment with the YAP inhibitor verteporfin decreased both YAP and BMI1 levels controlling HCC progression [61]. Also, in HBV-positive HCC cases, the preS2 domain of C-terminal truncated middle surface protein, another transactivator encoded by HBV, upregulated TAZ by suppressing miR338-3p fueling HCC proliferation [62].

A few conflicting studies reported Hippo signaling activation and decreased YAP in HCV E2 protein-treated/HCV nonstructural protein 4B (NS4B)-overexpressing normal human hepatocytes [63, 64].

### Hippo pathway and alcoholic/nonalcoholic steatohepatitis

While the incidence of HBV/HCB-driven HCC has declined, the proportion of liver cancer patients affected by NAFLD and NASH is rapidly increasing [65]. NAFLD is a chronic inflammatory syndrome often associated with metabolic disorders including obesity and type 2 diabetes mellitus that can progress to NASH, a severe disease associated with inflammation and fibrosis. NAFLD represents the most common chronic liver disease with a worldwide prevalence of 25% [66].

Consistent with the profound interconnections of Hippo signaling with metabolic processes both in normal and tumor backgrounds, several mechanisms of Hippo pathway dysregulation have been described in steatoepathitis and fatty liver disease. In NAFLD patient tissues and NASH mouse model, YAP was upregulated in the nuclei of reactive ductal cells (RDCs) responsible for production of pro-fibrogenic factors and expansion of YAP + RDCs cell population correlated with myofibroblast accumulation and fibrosis [67]. Consistent with a role in the progression from benign steatosis to fibrosis-associated steatohepatitis, TAZ expression was higher in murine NASH liver hepatocytes than in liver affected by benign steatosis. Importantly, in a mouse model of steatosis, the overexpression of TAZ in hepatocytes promoted fibrosis and NASH through upregulation of Indian hedgehog (IHH), a fibrogenic genes activating factor. Conversely, knockdown of hepatocyte TAZ in murine models of NASH reversed hepatic inflammation and fibrosis but not steatosis [68]. Similarly, fatty acid overload in hepatic cells of a NASH mouse model upregulated the obesity-associated junctional cadherin 5 associated protein (JCAD) that in turn inhibited LATS2 prompting YAP-mediated progression to liver cancer [69]. The Hippo regulatory kinase SAV1 was identified as an early driver of tumor development in two mouse models of NAFLD-HCC progression [liver-specific Phosphatase and tensing homolg (PTEN) KO and high-fat diet-fed mice], through Sleeping Beauty transposon mutagenesis screens. Supporting a crosstalk between the Hippo and PI3K pathways in NAFLD-HCC progression, liver-specific deletion of SAV1 promoted fibrogenesis and accelerated hepatocarcinogenesis in PTEN KO mice while SAV1 and PTEN are downregulated in nonviral HCC cases from the TCGA [70]. Furthermore, bile acids (BAs), whose metabolism dysregulation has been linked to steatosis and fibrosis in NAFLD patients, activate YAP via the scaffold protein IQGAP1 [71].

Alcohol consumption is an established risk factor for HCC accounting for 20% (in Southern European countries) to 63% (in Eastern European countries) of cases [72]. In alcohol-associated liver disease, upregulated FK506-binding protein 51 (FKBP5) inhibits MST1 leading to YAP-TEAD1-dependent transcription of CXC motif chemokine ligand 1 (CXCL1), a neutrophil chemoattractant causing hepatic inflammation [73].

### Hippo pathway and liver fibrosis

Disruption of Hippo signaling has been reported early during liver fibrosis, a common condition associated with HBV/HCV infection, alcohol abuse and liver steatosis [74]. Firstly, high YAP/TAZ expression levels correlate with this chronic disease [67, 75]. In mice models of liver fibrosis, YAP/TAZ activity in hepatocytes result in induction of Cysteine-rich angiogenic inducer 61 (CYR61) expression. CYR61 is a direct YAP/TAZ target and acts as a chemokine able to recruit macrophages sustaining liver fibrosis and immune response. Accordingly, YAP KO mice showed an impairment in macrophages recruitment and reduced fibrosis/inflammation [76]. Then, YAP is required for the activation of hepatocellular stellate cells (HSCs) that drive liver fibrosis [77]. During this process, mechanotransduction dysregulation results in excessive remodeling of the extracellular matrix (ECM) in HSCs causing their differentiation into highly proliferative and fibroblastic myofibroblasts [78, 79]. In mice, carbon tetrachloride (CCl_4_)-induced hepatocellular injury induces YAP translocation to the nuclei of HSCs and activation of transcriptional programs finally resulting in matrix and cytoskeleton remodeling and cell proliferation. In turn, HSCs ECM stiffening triggers YAP activation in a feedback loop that sustains mechanotransduction-mediated proliferation and survival [20, 77]. YAP knockdown or pharmacological inhibition with verteporfin prevents HSCs activation and fibrogenesis. Conversely, inhibition of upstream Hippo pathway components allows YAP stability and HSCs activation. Mannaerts and colleagues also demonstrated that YAP aberrant activation is an early event during fibrosis development, as YAP target genes ANKRD11 and CTGF, which are key components of fibrotic processes, are activated earlier than established markers of HSCs activation [77, 80]. In agreement, YAP inhibition attenuated liver fibrosis in CCl_4_-induced liver fibrosis mouse model [81, 82].

A wealth of evidence supports the driving role of Hippo pathway in linking liver fibrosis and cancer: (1) the functional crosstalk of Hippo pathway with TGF-β and NOTCH pro-oncogenic pathways has been reported early during liver fibrosis [32, 83–85]; and (2) YAP and TAZ support chronic inflammation, which is a major inducer of liver fibrosis, and liver cancer. In human liver tumors, TAZ expression was associated with secretion of pro-tumorigenic inflammatory cytokines interleukin-6 (IL-6) and CXCL1 [86]. Similarly, in mice, liver-specific deletion of MST1/2 and SAV1 is associated with elevated expression of IL-6 and Tumor necrosis factor-α (TNF-α) [36]. Also, murine liver-specific deletion of MST1/2 resulted in YAP-mediated chemoattractant protein 1 (MCP1) upregulation and massive infiltration of macrophages sustaining a protumoral microenvironment and liver overgrowth. YAP removal restored normal liver growth indicating the key role of Hippo signaling in restricting liver growth and carcinogenesis [87]. Accordingly, YAP activation requires inflammation-related signals to promote hepatocytes growth [88].

Overall, multiple mechanisms of Hippo pathway dysregulation have been described in chronic inflammation and fibrosis preceding liver carcinogenesis, providing the rationale for exploring YAP/TAZ targeting as a tumor-preventing strategy.

## Hippo pathway dysregulation on the way to esophageal and gastro-esophageal junction cancer

In EC patients, Hippo pathway dysregulation resulting in YAP activation/upregulation is associated with aggressive clinicopathological features and adverse clinical outcomes [89]. Preclinical studies revealed a critical role for YAP in dictating cancer stem cell (CSC)-like properties of EC cells [90–92]. Several upstream cues have been reported that account for YAP/TAZ aberrant activation including microRNAs [93, 94], proteins controlling YAP ubiquitination such as SHARPIN, PARK2 and RACO-1 [95–97], and YAP-interacting chromatin remodeling factors [98].

**Fig. 3:**
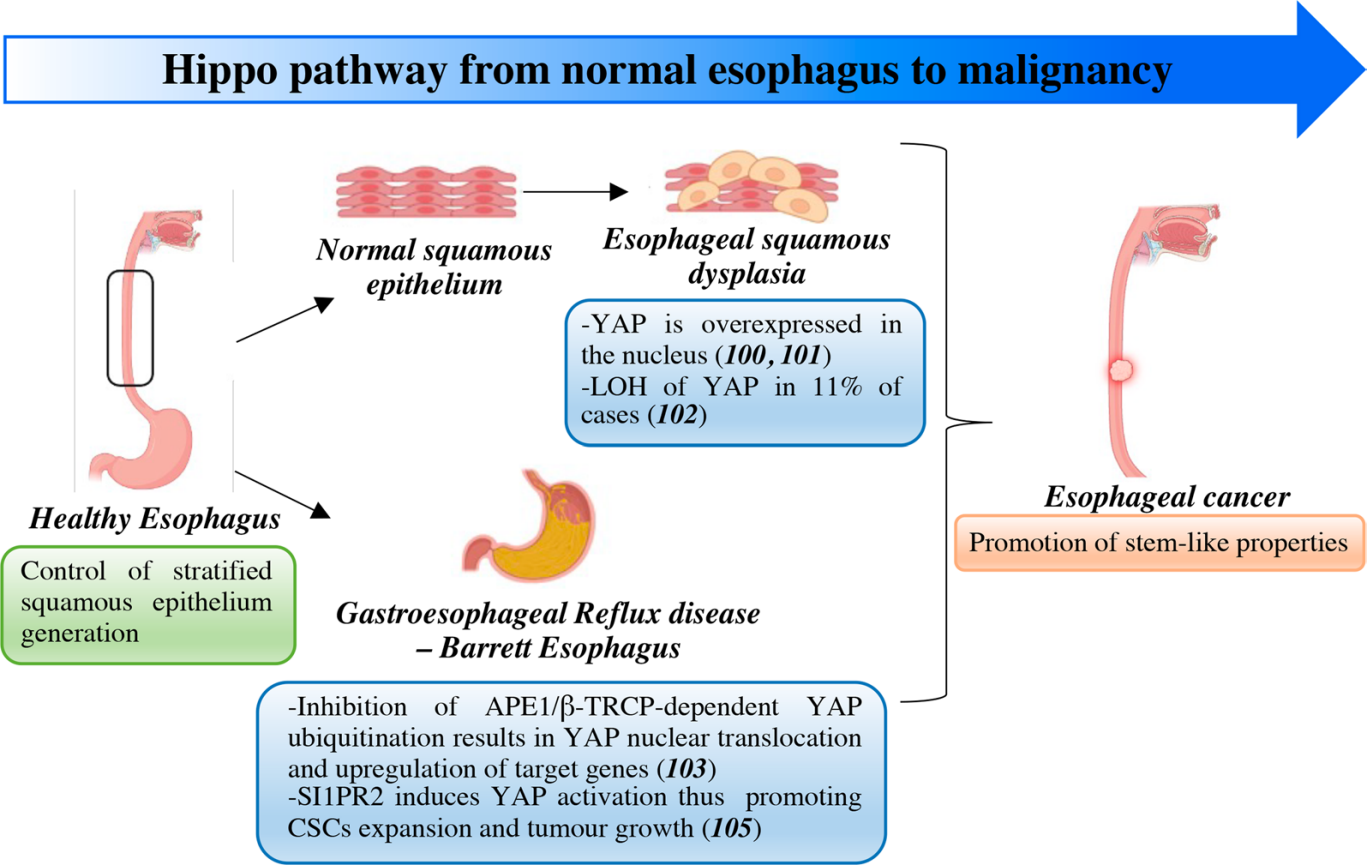
Hippo pathway dysregulation from esophageal precursor lesions to malignancy*.* Mechanisms contributing to dysfunctional Hippo signaling from normal to neoplastic esophagus across gastroesophageal reflux disease-Barrett esophagus/dysplasia. APE1: apurinic/apyrimidinic endonuclease 1; CSC: cancer stem cell; LOH: loss of heterozygosity; S1PR2: sphingosine 1-phosphate receptor 2; β-TRCP: β-transducin repeat-containing protein; YAP: Yes-associated protein

Predisposing conditions to tumors of the esophagus/gastroesophageal junction encompass esophageal squamous dysplasia (ESD), gastroesophageal reflux disease (GERD), which is the main risk factor associated with Barrett’s esophagus (BE), and infectious esophagitis. These conditions are all characterized by chronic inflammation [99]. A few studies have been carried out that aim to characterize the molecular features of esophagus preneoplastic lesions. Nevertheless, dysregulated Hippo signaling as denoted by high nuclear YAP has been described in high-grade dysplastic esophagus, associated or not with BE [100, 101]. In addition, whole-genome sequence analyses conducted on esophageal squamous cell carcinoma and dysplastic patient tissues revealed loss of heterozygosity of YAP1 as a shared event occurring in 12% and 11% of cases respectively [102]. YAP was also found to be upregulated in preclinical models of BE cells. Exposure of BE cells to acidic bile salts, exploited as a mimicry of GERD-associated reflux condition, induced the DNA-repair enzyme APE1-mediated inhibition of YAP ubiquitination by the E3 ubiquitine ligase β-TRCP, YAP nuclear accumulation and upregulation of its target genes, and YAP-dependent induction of stem-like features [103]. Other mechanisms of BA-induced activation of YAP have been described. Long-term treated esophageal keratinocytes showed induction of YAP and stem cell markers in association with pro-inflammatory signatures [104]. In addition, BA exposure promoted CSC expansion and invasive growth of esophageal adenocarcinoma cells through sphingosine 1-phosphate receptor 2 (S1PR2)-mediated activation of YAP [105] (Fig. 3).

## Hippo pathway dysregulation on the way to pancreatic cancer

Although YAP and TEAD are implicated in the regulation of pancreatic multipotent progenitor cells [106], studies investigating the role of the Hippo pathway in pancreatic development and homeostasis were not as informative as in other organs. However, it was clearly demonstrated that Hippo kinases play key roles in maintaining pancreatic acinar differentiation in mice [107].

YAP overactivity is considered an important oncogenic avenue in PDAC and studies carried out in mice have helped elucidating the oncogenic role of YAP/TAZ in pancreatic tumorigenesis [108–112]. Also, YAP/TAZ are involved in PDAC metastatization [113, 114] and chemoresistance [115].

Among preneoplastic disorders of the pancreas are: (1) sporadic chronic pancreatitis (CP), an inflammatory disease that is mainly due to high alcohol consumption; (2) hereditary CP, a relatively rare autosomal dominant disorder associated with a higher risk of cancer development than the sporadic form [116]; (3) pancreatic intraepithelial neoplasia (PanIN) arising in pancreatic ducts, which progresses from low-grade to high-grade and finally evolving to PDAC [117]; (4) cystic lesions like intraductal papillary mucinous neoplasms (IPMNs) or mucinous cystic neoplasms (MCNs) [118]; and (5) intraductal tubulopapillary neoplasm (ITPN), a rare premalignant condition [119].

A distinctive tract of eventual CP development into PDAC is represented by acinar-to ductal metaplasia (ADM), a reprogramming process during which pancreatic acinar cells differentiate into ductal-like cells [120]. Studies conducted in mice have been instrumental in defining KRAS activating mutation as a key ADM-initiating event. While selective expression of KRAS^G12D^ in embryonic cells of acinar lineage results in ADM progressing to invasive PDAC, KRAS^G12D^ specifical targeting to the adult mice pancreatic cells induces the onset of invasive PDAC only when an underlying, chemically-induced, CP background is present [120]. In an attempt to define how KRAS mutation cooperate with CP to promote progression to PDAC, Gruber and colleagues exploited mouse models of chemically-induced and KRAS^G12D^-induced pancreatitis and found that acinar cells undergoing ADM showed high levels of nuclear YAP and TAZ. Accordingly, in human tissues, both YAP and TAZ are highly expressed in pancreatic stellate cells (PSCs) which are activated in the microenvironment by CP-associated damaged acinar cells and dominate the fibrotic and inflammatory microenvironment associated with this condition [121].

Several studies have been conducted to elucidate whether YAP/TAZ activation is involved at an early stage during ADM-PDAC progression. Zhang and colleagues reported that YAP deletion in a KRAS^G12C^ mouse model did not affect ADM and early PanIN, while totally impairing late-stage PanIns and PDAC [122]. Later, by using a different KRAS^G12C^ mouse model, Gruber and colleagues reported that ectopic YAP/TAZ activation in mouse acinar cells was sufficient to induce ADM through activation of the Janus kinase (JAK)/Signal transducer and activator of transcription 3 (STAT3) signaling pathway. Co-deletion of YAP and TAZ impaired KRAS^G12C^-induced formation of ADM lesions [123]. A wealth of evidence is concordant with an early involvement of Hippo pathway disruption. Selective deletion of LATS1/LATS2 in pancreatic acinar cells of adult mice is sufficient to cause YAP/TAZ-dependent acinar cell atrophy and stimulation of PSCs activation that is followed by infiltration of immune cells. Importantly, activation of PSCs preceded ADM, suggesting that Hippo disruption in acinar cells induces stromal activation as an early event [124]. Mechanistically, YAP coordinates ADM initiation through a network of functional interactions. The crosslink with polymerase-associated factor 1 (PAF1) in metaplastic ducts of mouse cereulin- or KRAS^G12C^-induced ADM and in human PC cells induces SRY-box transcription factor 9 (SOX9) transcription and stemness. YAP targeting by verteporfin inhibited ADM and PC cell growth [125]. In addition, the functional interaction with PI3K pathway is involved in this process. While both avenues were deregulated in CP mouse models, the pancreas-specific depletion of PTEN and SAV1 was sufficient to induce CP in mice. In acinar cell models, double knockdown of PTEN and SAV1 induced CTGF-mediated ADM [126]. Finally, YAP ablation in KRAS^G12C^ mouse models of pancreatic tumor induced tumor regression and lineage switching from ductal cells to acinar cells, reverting the phenomena of ADM [111].

In contrast to aggressive PDAC developing from CP-PanIN process, cancers arising from cystic lesions are characterized by prolonged latency with the vast majority of these lesions not progressing. In GNAS^R201C^- KRAS^G12C^ transgenic mice developing differentiated IPMN-like cystic lesions, Hippo cascade was found to be actively signaling. Accordingly, human GNAS-mutated IPMN tissues showed YAP cytoplasmic localization [110]. The lack of aberrant Hippo signaling was independently confirmed in mouse models of IPMN triggered by a different genetic background, providing a potential explanation of the indolent biology underlying IPMN-PDAC progression [109]. Finally, TAZ activation was detected in human ITPN tissues and in a mouse model of ITPN-PDAC progression, obtained through double KO of the tumor suppressor PTEN and AT-Rich Interaction Domain 1A (ARID1A) in pancreatic ductal cells [109].

**Fig. 4:**
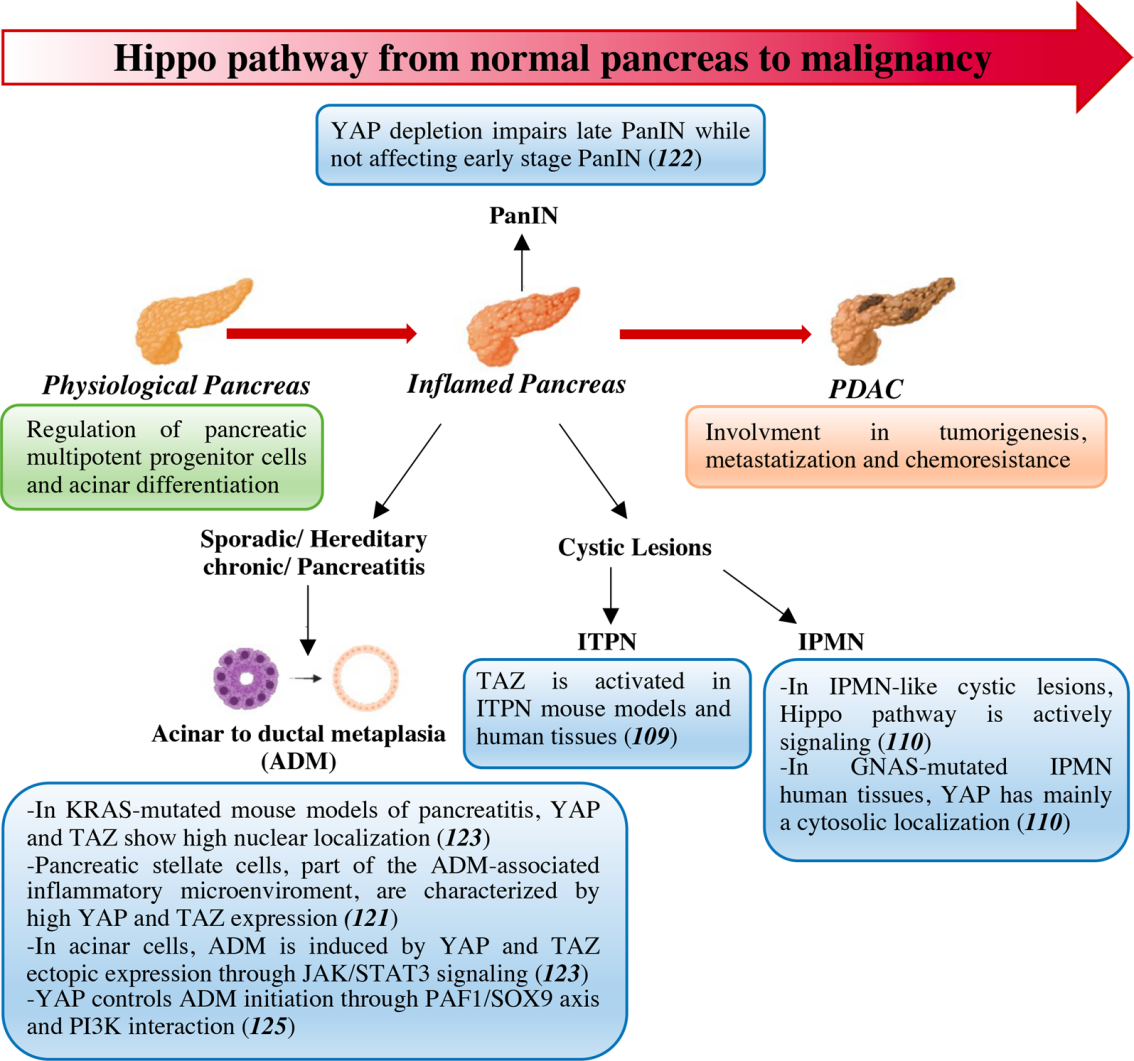
Hippo pathway dysregulation from pancreas precursor lesions to malignancy*.* YAP/TAZ aberrant activation is an early event in sporadic and hereditary pancreatitis, pancreatic intraepithelial neoplasia, mucinous cystic lesions and intraductal tubulopapillary neoplasm. ADM: acinar-to ductal metaplasia; CP: chronic pancreatitis; GNAS: guanine nucleotide binding protein, alpha stimulating;IPMN: intraductal papillary mucinous neoplasm; ITPN: intraductal tubular papillary neoplasm;JAK: janus kinase; KRAS: Kirsten rat sarcoma virus; PAF1: polymerase-associated factor 1; PanIN: pancreatic intraepithelial neoplasia; PDAC: Pancreatic ductal adenocarcinoma; PI3K: phosphoinositide 3-kinase; SOX9: sex-determining region Y-box 9; STAT: signal transducer and activator of transcription; TAZ: Transcriptional co-activator with PDZ-binding domain; YAP: Yes-associated protein

Overall, these studies clearly define that YAP/TAZ activation is an early event in preneoplastic lesions of the pancreas and it is required for progression to carcinogenesis (Fig. 4).

## Hippo pathway dysregulation on the way to colorectal cancer

The Hippo pathway plays crucial roles in normal development, regeneration and carcinogenesis of the intestinal compartment [16]. In the mouse intestine, YAP is mainly expressed in crypts where it is involved in stem cells expansion and renewal. Here, in response to injury, YAP reprograms the intestinal stem cells through interaction with the Wnt/β-catenin signaling pathway and induction of regenerative programs [30, 127].

Dysregulation of the Hippo pathway and hyperactivation of YAP/TAZ are common traits observed in most CRC patients and are fueled by multiple mechanisms including crosstalk with Wnt/β-catenin pathway [128], recurrent Adenomatous Polyposis Coli (APC) loss-of function mutations [127] and deregulation of inhibitory pathways [129–132]. Similarly to other solid tumors, YAP/TAZ control CCR progression, metastatization and therapeutic resistance through various mechanisms [133–137].

**Fig. 5:**
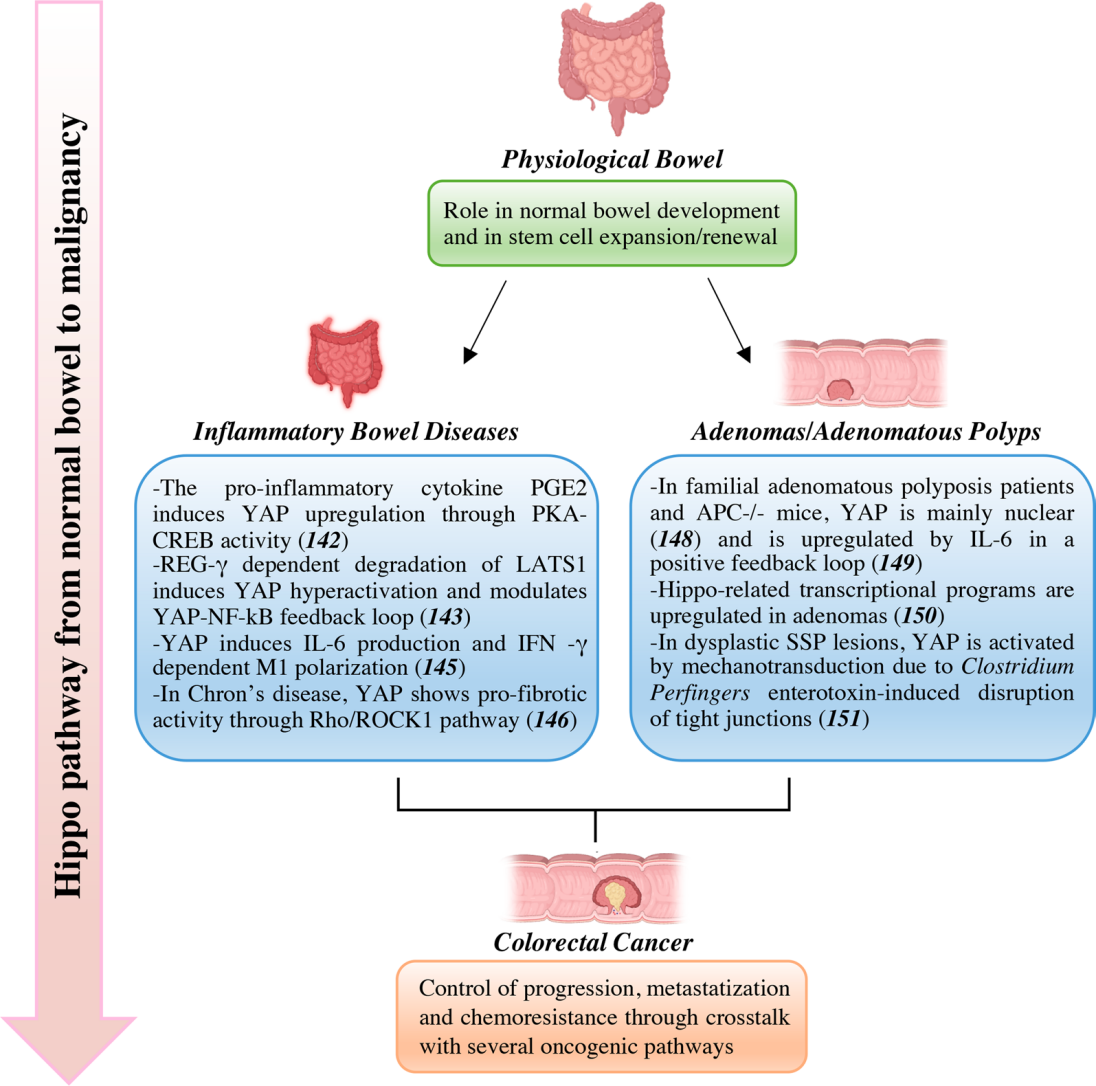
Hippo pathway dysregulation from bowel precursor lesions to malignancy*.* Mechanisms of aberrant YAP/TAZ activation associated with inflammatory bowel disease and adenomas/adenomatous polyps. APC: adenomatous polypolis coli; CREBP: cAMP response element-binding protein; IL-6: interleukin 6/; INF-γ: interferon-gamma; LATS1: large tumor suppressor 1; NF-κB: nuclear factor kappa-light-chain-enhancer of activated B cells; PGE2: prostaglandin E2; PKA: protein kinase A; REG-γ: proteasome activator subunit 3; ROCK1: Rho-associated protein kinase 1; SSP: sessile serrated polyps; YAP: Yes-associated protein

Since 1990s, incidence and mortality rates of CRC have been progressively increasing particularly among younger adults, paralleled by prevalence of well-known risk factors including smoking, red and processed meat consumption, obesity and alcohol use [138]. Recent studies have estimated diet and lifestyle factors to contribute to 50–60% of CRC cases in the United States, possibly through complex metabolic, inflammatory and gut microbiota-related mechanisms [139–141]. Chronic inflammations is the underlying condition of inflammatory bowel disease (IBD) which comprises Chron’s disease (CD) and ulcerative colitis (UC). Finally, adenomas and adenomatous polyps are the ultimate precursors of almost all CRCs. Mechanisms of aberrant YAP/TAZ activation associated with these conditions are described below and summarized in Fig. 5.

### Hippo pathway and inflammatory bowel disease

Mouse models of dextran sulfate sodium (DSS)-induced colitis have helped elucidating the connections of Hippo signaling with inflammatory processes as well as the involvement of YAP in both IBD pathogenesis and IBD-CRC progression (Fig. 5). Firstly, YAP is induced by the inflammatory cytokine Prostaglandin E2 (PGE2) to regulate colon regeneration and carcinogenesis. Mechanistically, the inflammation-induced bond of PGE2 and prostaglandin E receptor 4 gene (EP4) activates Protein Kinase A (PKA) and CREB, resulting in YAP transcription. Moreover, this process is fueled by a positive feedback loop, in which YAP-TEAD interaction mediates the activation of Prostaglandin-endoperoxide synthase 2 gene (PTGS2 or COX2) and EP4, both components of the PGE2 pathway. Accordingly, PGE2 has been found to be upregulated in CRC murine models and in tissues from CRC patients and colitis-associated tumors [142]. An additional YAP-activating mechanism is mediated by the Proteasome activator Subunit 3 (REG-γ). Evidence from in vitro and in vivo models indicates that REG-γ sustains colon inflammation enhancing cancer susceptibility by two mechanisms: (i) overactivation of YAP mediated by LATS1 degradation; and (ii) modulation of the positive feedback loop between YAP and Nuclear factor-kappa B (NF-κB), a master regulator of inflammation [143].

Accordingly, YAP overexpressing mice are more susceptible to cancer development in response to DSS treatment, due to crosstalk of YAP with β-catenin and STAT3 signaling which activate transcriptional programs fueling stemness and proliferation [30, 144]. YAP was reported to aggravate IBD by fueling aberrant M1/M2 macrophage polarization, which is a distinctive trait of the disease. Mechanistically, YAP impairs IL-4/IL-13-induced M2 macrophage polarization, which have anti-inflammatory roles, while promoting Interferon-gamma-induced activation of M1 macrophage and inflammatory IL-6 production. In mice with DSS-induced colitis, YAP knockout rescued M2 macrophage polarization and production of anti-inflammatory cytokines, alleviating colitis [145].

YAP/TAZ exert profibrotic function in CD which is characterized by ECM deposition on the mucosa resulting in fibrosis and intestinal obstruction. Rho-Rho-associated protein kinase 1 (ROCK1) signaling pathway-activated YAP and TAZ are enriched in fibroblasts isolated from the stenotic intestines of CD patients where they promote the expression of profibrotic genes. ROCK1 inhibition reduced YAP/TAZ and profibrotic genes expression in isolated fibroblasts and showed antifibrotic effect in mouse models of CDD-induced colitis [146].

### Hippo pathway and adenomas/adenomatous polyps of the intestine

While germline mutation of the APC tumor suppressor gene is the underlying condition for familial adenomatous polyposis (FAP), APC somatic mutations occur in approximately 80% of all CRC cases [147]. In addition to its well known role as a negative regulator of β-catenin, APC has been reported to inactivate YAP both in a β-catenin destruction complex-dependent and independent manner [128, 148]. Accordingly, nuclear accumulation of YAP was observed in intestinal adenomas of APC^Min/+^ mice and in tubular adenoma tissues of FAP patients [148]. Similarly, in APC^−/−^ mouse small intestine-derived organoids, expression of the cytokine co-receptor IL-6 signal transducer (IL-6ST/gp130) is upregulated resulting in the simultaneous activation of STAT3 and Src family kinases (SFKs)-YAP axis. YAP in turn activates IL-6ST transcription sustaining a positive feedback loop which promotes initiation of colorectal tumorigenesis [149].

Chen and colleagues exploited single cell-RNA sequencing to define the cellular origins of the two most common precancerous lesions of human colorectum: adenomas and sessile serrated polyps (SSP). The authors uncovered that adenomas develop from stem cell expansion programs while SSP arise from metaplastic processes and revealed enrichment of Hippo-related programs only in adenomas [150]. However, a study conducted on progressive stages of SSP clarified the involvement of YAP only in dysplastic SSP lesions (SSP-D) immediately preceding CRC development. Human SSP-D tissues but not SSP specimens were characterized by *Clostridium perfringes* enterotoxin (CPE) expression and YAP activation. Mechanistically, in rat intestinal epithelial cells, CPE targeted the tight junction protein claudin-4 (CLDN4) to disrupt tight junction resulting in YAP activation by mechanotransduction [151].

## Conclusions

The Hippo pathway is a key regulator of cell proliferation and organ growth both in invertebrates and mammals. Investigations over the last decades have well documented pathway involvement in processes such as liver and intestinal regeneration, stem cell reprogramming and tissues homeostasis. YAP/TAZ-dependent programs of regeneration/proliferation are rapidly activated in response to signals released during inflammation and fibrosis, common traits of premalignant lesions of GI tumors. However, mouse models of chemically-induced inflammation revealed that sustained signaling such as in chronic diseases/conditions may unlock the oncogenic potential of YAP/TAZ transcriptional programs triggering malignancy. In agreement, aberrant activation of YAP/TAZ effectors and/or loss of function of upstream Hippo kinases are distinctive traits of GI tumors that represent a major cause of cancer-related mortality worldwide. Further supporting the critical role of YAP/TAZ in promoting premalignant to malignant transformation, the few benign lesions in which Hippo signaling is not implicated are characterized by prolonged latency.

Currently, several Hippo-YAP targeting therapies are under exploration in phase II and III clinical trials enrolling GI cancer patients, that are extensively addressed elsewhere [152]. However, premalignant YAP/TAZ aberrant activation provides the rationale for exploring YAP/TAZ targeting as a tumor-preventing strategy. Studies exploring Hippo targeting at the precancerous stage may add knowledge on the involvement of the Hippo pathway in the evolutionary trajectories tying precancerous lesions to GI tumors. In this perspective, a further consideration regards those tumors characterized by low survival rates, such as PDAC, where early identification and treatment of pancreatic precursor lesions, despite currently difficult, would be beneficial in improving survival rates.

An increased comprehension of the biology of Hippo dysregulation in GI precancerous lesions is crucial. Indeed, while large evidence has contributed to delineate the functional networks of connections between the Hippo axis and several cancer-related pathways in initiating and driving GI tumors onset and progression, several aspects of YAP/TAZ activation prior to GI carcinogenesis need to be addressed. For instance, the gut microbiota plays a key role in inflammatory bowel disease (IBD) that is associated with increased risk of cancer. Although YAP expression in macrophages is thought to alter the gut microbiota thus contributing to IBD, the precise mechanisms through which the Hippo pathway contributes to IBD remain largely unknown. A deeper knowledge of Hippo interactions with surrounding microbiota and microenvironment and how these crosslinks are shaped at the level of different cell types during cancer evolution will be instrumental in better framing YAP/TAZ orchestration of pro-tumorigenic processes.

## Data Availability

Not applicable.

## Abbreviations

ADM: Acinar-to ductal metaplasia
AMPK: Adenosine monophosphate-activated protein kinase
ANKRD11: Cardiac ankyrin repeat protein
APC: Adenomatous polypolis coli
APE1: Apurinic/apyrimidinic endonuclease 1
ARID1A: AT-rich interaction domain 1A
ASH: Alcoholic steatohepatitis
ATF4: Activating transcription factor 4
BA: Bile acid
BE: Barrett’s esophagus
BMI1: B lymphoma Mo-MLV insertion region 1 homolog
cAMP: Cyclic adenosine monophosphate
CCl_4_: Carbon tetrachloride
CD: Chron’s disease
CLDN4: Claudin-4
COX2: Cyclooxygenase 2
CP: Chronic pancreatitis
CPE: *Clostridium perfringes* Enterotoxin
CRC: Colorectal cancer
CREB: CAMP response element-binding protein
CSC: Cancer stem cell
CTGF: Connective tissue growth factor
CXCL1: CXC motif chemokine ligand 1
CYR61: Cysteine-rich angiogenic inducer 61
DSS: Dextran sulfate sodium
EC: Esophageal carcinoma
ECM: Extracellular matrix
EP4: Prostaglandin E receptor 4 gene
ESD: Esophageal squamous dysplasia
FAP: Familial adenomatous polyposis
FKBP5: FK506-binding protein 51
GC: Gastric cancer
GERD: Gastroesophageal reflux disease
GI: Gastrointestinal
GNAS: Guanine nucleotide binding protein: alpha stimulating
GPCR: G-protein-coupled receptors
GTP: Guanosine triphosphate
HBsAg: HBV surface antigen
HBV: Hepatitis B virus
HBx: Hepatitis B virus X protein
HCC: Hepatocellular carcinoma
HCV: Hepatitis C virus
HDM2: Human double minute 2
HSC: Heaptocellular stellate cell
IBD: Inflammatory bowel disease
IHH: Indian hedgehog
IL-4/6/13: Interleukin 4/6/13
IL-6ST/gp130: IL-6 signal transducer
IPMN: Intraductal papillary mucinous neoplasm
IQGAP1: IQ motif containing GTPase activating protein 1
ITPN: Intraductal tubular papillary neoplasm
JAK: Janus kinase
JCAD: Junctional cadherin 5 associated
KO: Knock-out
KRAS: Kirsten rat sarcoma virus
LATS1/2: Large tumor suppressor 1 and 2
MCN: Mucinous cystic neoplasm
MCP1: Monocyte chemoattractant protein-1
MOB1A/B: MOB kinase activator 1A and 1B
MST1/2: Serine/threonine kinases sterile 20-like kinase 1 and 2
NAFLD: Nonalcoholic fatty liver disease
NASH: Nonalcoholic steatohepatitis
NF-κB: Nuclear factor kappa-light-chain-enhancer of activated B cells
NOTCH: Neurogenic locus notch homolog protein
NS4B: Nonstructural protein 4B
PAF1: Polymerase-associated factor 1
PanIN: Pancreatic intraepithelial neoplasia
PARK2: Parkin 2
PDAC: Pancreatic ductal adenocarcinoma
PGE2: Prostaglandin E2
PI3K: Phosphoinositide 3-kinases
PKA: Protein kinase A
PSC: Pancreatic stellate cell
PTEN: Phosphatase and tensin homolog
PTGS2: Prostaglandin-endoperoxide synthase 2 gene
RACO-1: RING domain-containing protein RACO-1
RDC: Reactive ductal cell
REG-γ: Proteasome activator subunit 3
ROCK1: Rho-associated protein kinase 1
RUNX1/2: Runt-related transcription factor 1/2
SAV1: Salvador homolog 1
SCD1: Stearoyl-CoA desaturase-1
SFK: Src family kinase
SHARPIN: SHANK associated RH domain interactor
SHH: Sonic hedgehog
SMAD: Small mother against decapentaplegic
SOX9: Sex-determining region Y-box 9
S1PR2: Sphingosine 1-phosphate receptor 2
SSP: Sessile serrated polyps
STAT3: Signal transducer and activator of transcription 3
TAZ: Transcriptional co-activator with PDZ-binding domain
TBX5: T-box transcription factor 5
TCGA: The Cancer Genome Atlas
TEAD1/4: TEA domain-containing sequence-specific transcription factors 1/4
TGF-β: Transforming growth factor beta
TNF-α: Tumor necrosis factor-α
β-TRCP: β-Transducin repeat-containing protein
UC: Ulcerative colitis
YAP: Yes-associated protein
