# Correction to: Role of Hippo pathway dysregulation from gastrointestinal premalignant lesions to cancer

**DOI:** 10.1186/s12967-024-05161-3

**Published:** 2024-05-03

**Authors:** Giulia Schiavoni, Beatrice Messina, Stefano Scalera, Lorenzo Memeo, Cristina Colarossi, Marzia Mare, Giovanni Blandino, Gennaro Ciliberto, Giulia Bon, Marcello Maugeri-Saccà

**Affiliations:** 1 Clinical Trial Center, Biostatistics and Bioinformatics Unit, Department of Research, Diagnosis and Innovative Technologies, IRCCS Regina Elena National Cancer Institute, Rome, Italy; 2 SAFU Laboratory, Department of Research, Advanced Diagnostic, and Technological Innovation, IRCCS Regina Elena National Cancer Institute, Rome, Italy; 3 Pathology Unit, Mediterranean Institute of Oncology, Viagrande, Italy; 4 Medical Oncology Unit, Mediterranean Institute of Oncology, Viagrande, Italy; 5 Department of Biomedical, Dental, Morphological and Functional Imaging Sciences, University of Messina, Messina, Italy; 6 Translational Oncology Research Unit, Department of Research, Diagnosis and Innovative Technologies, IRCCS Regina Elena National Cancer Institute, Rome, Italy; 7 Scientific Directorate, IRCCS Regina Elena National Cancer Institute, Rome, Italy; 8 Cellular Network and Molecular Therapeutic Target Unit, Department of Research, Diagnosis and Innovative Technologies, IRCCS Regina Elena National Cancer Institute, Via Elio Chianesi 53, 00144 Rome, Italy; 9 Division of Medical Oncology 2, IRCCS Regina Elena National Cancer Institute, Rome, Italy

**Correction to: Journal of Translational Medicine (2024) 22:213**

10.1186/s12967-024-05027-8

Following publication of the original article [1], we have been notified that the Funding note was published incorrectly.

It is now:

Dr. Maugeri-Saccà is supported by the Italian Association for Cancer Research (AIRC) under MFAG 2019 (project identification: 22940) and the Italian Ministry of Health (MoH) (project identification: GR-2016?02362025).

It should be:

This work was financially supported through funding from the institutional “Ricerca Corrente” granted by the Italian Ministry of Health.
